# Supplementary material for: Early resumption of postpartum sexual intercourse and its associated risk factors among married postpartum women who visited public hospitals of Jimma zone, Southwest Ethiopia: A cross-sectional study
Source: PLoS One. 2021 Mar 29;16(3):e0247769. doi: 10.1371/journal.pone.0247769 (PMC8007007; doi:10.1371/journal.pone.0247769)
Supplement: S1 Questionnaire — (DOCX) [file pone.0247769.s001.docx]

**ANNEX THREE: QUESTIONNAIRES IN ENGLISH VERSION**

**Instruction-** Dear data Collector, what is expected from you is to encircle for multiple choices questions and write an appropriate response for some open ended questions on the given space and please not forget to check skipping.

Questionnaire code _________________ Hospital code _________________

Name of data collector -------------------------Data of interview---------------------------------

**Section I. Information on socio demographic characteristics (circle the correct answer/fill in space provided).**:

| No | Questions | Response options | Skip |
| --- | --- | --- | --- |
|  | How old are you? | ______________in years |  |
|  | What is your ethnic group? | 1. 1.Oromo 2. 2.Dawro 3. 3.Keffa 4. 4.Gurage 5. 5. Others (Specify)……….. |  |
|  | What is your residence? | - - - 1. Urban       2. Rural |  |
|  | What is your religion? | 1. Muslim 2. Orthodox Christian 3. Protestant Christian 4. Catholic 5. Others (specify)…………. |  |
|  | How many wife your husband has? | ___________________in number |  |
|  | Duration of living together with Husband? | _____________________in year(s) |  |
|  | What is your occupation? | 1. House wife 2. Self-employed/private 3. Gov’t employed 4. Student 5. Others, specify---------------- |  |
|  | What is your educational level? | - - - 1. Can’t read and write       2. Primary(1-8)       3. Secondary(9-12)       4. College/university graduate |  |
|  | How much is your income per month? | ___________________ETB |  |

**Section 2: Reproductive and Maternal health service use-related characteristics (circle the correct answer/fill in space provided)**

| S.No | Questions | | | Response options | | | Skip |
| --- | --- | --- | --- | --- | --- | --- | --- |
|  | How many time(s) have you been pregnant? | | | _____________in number | | |  |
|  | If greater one on Q201, what was the time interval between the previous and the last pregnancy? | | | ______________in month | | |  |
|  | Did you have any history of abortion? | | | 1.Yes  2.No | | |  |
|  | If yes on Q203, how many abortion have you had? | | | _____________in number | | |  |
|  | How many time(s) have you given birth after 28 weeks of GA? | | | ______________in number | | |  |
|  | How many alive children do you have now? | | | _______________in number | | |  |
|  | Was your last pregnancy planned? | | | 1.Yes  2.No | | |  |
|  | Did you have ANC visit during last pregnancy? | | | 1.Yes  2.No | | |  |
|  | If yes on Q208, How many time(s)? | | | _____________________in number | | |  |
|  | Did you have any obstetric related complications during last pregnancy? | | | 1.Yes  2.No | | |  |
|  | If yes on Q210, what was it?tick for multiple response | | | 1.APH  2.Hypertensive disorders  3.PROM  4. Other, specify……………………. | | |  |
|  | Where did you give birth for the last delivery? | | | 1.Health center  2.Governmental hospital  3.Profitable private hospitals/clinics  4.At home | | |  |
|  | What was your mode of delivery? | | | 1. Vaginal delivery without episiotomy 2. Vaginally delivery with episiotomy/tear 3. Cesarean section | | |  |
|  | For how long, does your onset of labor lasts? | | | ________________in hour(s). | | |  |
| Section Three: Post natal care related factors (circle the response /fill in space provided). | | | | | | | |
|  | | Did you have PNC visit for the last birth? | 1.Yes  2.No | | |  | |
|  | | Have you had any post-partum related complication(s)? | 1.Yes  2.No | | | If no “skip” to Q 304 | |
|  | | If yes for Q 302, what was it? Tick for multiple response | 1.PPH  2.Puerparal sepsis  3.Hypertensive related disorders  4.othe,specify,,,,,,,,,,,,,, | | |  | |
|  | | What is your currently infant feeding practice? | 1.Exclusive breast feeding  2.Formula feeding | | |  | |
|  | | Did you resumed menstruation after recent birth ? | 1.Yes  2.No | | |  | |
|  | | Do you know any modern contraceptive methods? | 1.Yes  2.No | | | If no skip to Q 307 | |
|  | | If yes on Q 305,circle from the list? | 1.Pills  2 .Injectable  3.Implants  4.IUCD  5.Permanant methods  6.if other ,specify | | |  | |
|  | | Are you currently using any method of contraception? | 1. Yes 2. No | | |  | |
|  | | Which contraceptive methods you are using currently? | Pills | | |  | |
|  |  |  | Injectable | | |  | |
|  |  |  | Implant | | |  | |
|  |  |  | IUCD | | |  | |
|  |  |  | Other | | |  | |
| **Section Four: Sexual health during pregnancy and after birth related characteristics (circle the correct answer/fill in space provided).** | | | | | | | |
|  | | Did you practice any sexual intercourse during last pregnancy? | 1.Yes  2.No | | If no Go to Q 403 | | |
|  | | Which week of pregnancy did your last intercourse ends? | ---------------------In weeks | |  | | |
|  | | When did you resume sexual intercourse in weeks? | ----------------------In weeks | |  | | |
|  | | What was your reason for the resumption sexual intercourse? | 1. Husband demand sex  2.Jointly decision  3.If other(specify),,,,,,,, | |  | | |
|  | | Did you encounter any associated sexual morbidities during sexual intercourse resumption? | 1.Yes  2.No | | If no skip to 409 | | |
|  | | If “Yes” for Q 4, What have you encountered? Tick for multipe answers | 1. Dyspareunia  2.Lack of sexual desire  3.Genital tear  4.Vaginal bleeding  5.abnormal vaginal discharge  6. other, specify……………………… | |  | | |
|  | | Did you seek Health care for mentioned sexual problem? | 1.Yes  2.No | |  | | |
|  | | Have you received any sexuality education/advise about post partum sexual health? | 1. Yes  2 .No | |  | | |

ANNEX-FOUR: CONSENT FROM IN LOCAL VERSION/AFAAN OROMO

**AF-GAAFFII DUBARTOOTA DA’UMSAA BOODA GARA HOSPITAALA DHUFANIF QOPHAA’E**

**Kun unkaa waliigaltee**

Gaaffannoon kun dubartoota idattoof filtaman qofa kan illaallatudha .kaayyoon qoranno kanaa da’umsaa booda turtii hagami kassee wal-qunnamtii saalad a’umsaa booda itti eegalee fi sababiwwan saal-qunnamti eegalatan kan ilaaltuudha qaaccessuudha.oddeeffanoon sirra argadhu,hojii qorannoo kanaaf qofa kan ooluudha,kanaaf, bilisa ta’uun gaafannoo kana aakka naaf deebistu/tan kabajaansi/isiin gaafadha

Gaafatamuf eeyyamamadha ……………. A.Eeyyee B.Lakkii

MaqaaHospitaala,……………………

LakkKoodii……………………………..

Bu’a Gaafficha

1.Xumurameera

2.Hinargamneguyyagaafano

3.Nidide

4.Gartokkodeebisteeti/saniru

5.Kan biro yoota’ebareessi……………………

Galatoomaa!

ANNEX-IV: QUESTIONNAIRES IN LOCAL LANGUAGE VERSION/AFAAN OROMO

**Akkeekachisa-** Deebii sirri ta’ee itti marii fi waldura duuba gaafilee hindagatin

**Koodii** Gaaffatama_________________

**Kutaa1ffaa Gaaffiwwan Jireenya Hawasumma Ilaalatan (itti mari ykn bakka kenname irrati barreessi)**

| Lakk | Gaafilewan | Filanoo deebiiwani | ceesisaa |
| --- | --- | --- | --- |
|  | Umuriin/Ganna kee meeqa? | _________________waggaadhan |  |
|  | Sabni kee maali? | 1. 1.Oromoo 2. 2.Dawro 3. 3.Keffa 4. 4.Gurage 5. 5.Kan biro, ibsi……………………….. |  |
|  | Teessoon keeessa nessa? | 1.Magaalaa  2.Baadiyaa |  |
|  | Amantiin keessan maali? | 1 Muslimaa  2.Kiristaan orthodoksi  3.Kiristaan Protestanti  4.Kaatoliki  5.Kan biro yoota’e , ibsi…………. |  |
|  | Abban warra keeNiitii/Haadha warra meeqa qaba? | ______________lakkoofsan |  |
| 1. 6 | Wagga meeqaf abba warra kee waliin jiraata jirta? | ________________waggaadhan |  |
| 1. 7 | Hojiin kee maali? | 1.Haadha warra qofa  2.Hojii dhuunfa mataa koon qaba  3. Hojeettu motummatti  4.Barattuu  5.Kan biro yoota’e , ibsi…………. |  |
| 1. 8 | Sadarkan barumsa keeti maal fakkata? | 1.Hin Baranne  2.Sadarkaa tokkoffa (1-8)  3.Sadarkaa lamaffa(9-12)  4.Kolleejji/Yuunivarsitiixumureraa |  |
|  | Galiin kee Ji’an maal fakkata? | _________________birri itoopihaan ka’i |  |

Kutaa2ffaa Gaafilee wal-hormataa fayyaa ilaaltan (itti mari ykn bakka kenname guti )

| Lakk | Af-Gaaffiwwan | Filanoo deebiiwani | | Cesisa |
| --- | --- | --- | --- | --- |
|  | Meeqa ulfoofte beekta? | ___________Lakkoofsan | |  |
|  | Yoo tokko olta’e Gaffii 201ffaa irratti garagarumma ji’a meeqa jiddutti ulfa isa dhiyeenya ulfoofte? | ___________Ji’anka’i | |  |
|  | Ulfi sirra bahe beeka? | 1.Eeyye 2.Lakki | | G 205 |
|  | Eeyye yoo jette G204,irratti yeroo meeqa sira bahe? | ___________Lakkoofsan | |  |
|  | Meeqa deesse torbe 28 olitti? | __________Lakkoofsan | |  |
|  | Ijoollee meeqa qabda? | ___________Lakkoofsan | |  |
|  | Karoori ulfa kee isa amma maal fakkata? | 1.Karoorani  2.Karooran ala | |  |
|  | Hordoffi ulfa argatteta da’umsaa kee isa dhiyooti? | 1.eeyyee  2.lakkii | |  |
|  | Ulfa kee isa dhiyeenya,Meeqa deesse ? | ____________Lakkoofsan | |  |
|  | Rakkoolen yeroo ulfaa keeti si mudatan jiru? | 1 .Eeyyee2.Lakkii | |  |
|  | Yoo eeyyee jette Gaaffii 211, maal inni? | 1.dhangala’uu dhiiga da’umsan dura  2.Rakkoole dhibba dhiiga wajjiin walqabatan  3.Dhangaluu dhangala’a qaama salattin  4. Kan biro yoota’e ,ibsi…………. | |  |
|  | Da’umsa kee isa dhiyeenya essatti deesse? | 1.Buufata Fayyaa  2.Hospitalaa Motummatti  3.Ciliniikaa/Hospitalaadhuunfaatti  4.Mana kooti | |  |
|  | Haalli da’umsa keeti maal fakkataa? | 1.Rakko tokkomalee  2.Gargarsaa xiqqo murani hodhudhan qamaas  3.Gargarsaa murani hodhuu garaadhan | |  |
|  | Sa’aatti meeqa sitti fudhatee da’umsi kee in dhiyeenya deesse? | _______________sa’aatidhan | |  |
| Kutaa 3ffaa Gaaffilee fayyaa haadholii da,umsa booda fi karooraa maatii waliin walqabatan (itti mari ykn bakka kenname irrati barreessi) | | | | |
|  | Hordoffii taasifteeta erga deesse booda ? | 1.eeyyee  2.lakkii | |  |
|  | Rakkooleen da’umsa booda si mudatani beeku? | 1.eeyyee  2.lakkii | |  |
|  | Yoo eeyyee Jette Gaaffii 303f, maal inni? | 1.Dhangala’uu dhiiga da’umsa booda  2.Dhangaluu dhangala’a qaama salattin/  mallattodhukkubiagarsiisu  3.Rakkoole dhibba dhiiga wajjiin walqabatan  4. Kan biro yoota’e ,ibsi…………. | |  |
|  | Harmaa mucaa kee hoosisaa jirtaa? | 1.Harma haadha qofa  2.Nyaata dabaltaa fi harma haadha | |  |
|  | Laguun /Xuriin kee deebi’eera? | 1.Eeyye  2.Lakki | |  |
|  | Beekumsa waa’ee qusanoo matii ni qabda? | 1.eeyyee  2.lakkii | |  |
|  | Kamfaa beekta? | 1.kininii  2.lilimee  3.goga irree jala kanaa kawwamuu  4. Gadameesssa keessa kan kawwamu  5. Qosano umurii guutuu  6.Kan biro yoota’e ,ibsi…………. | |  |
|  | Qusanoo maatii itti fayyadama jirta? | 1.eeyyee  2.lakkii | |  |
|  | Isa kam fayyadama jirta? | 1.kininii  2.lilimee  3.goga irree jala kanaa kawwamuu  4. Gadameesssa keessa kan kawwamu  5.kondomii  6.Kan biro yoota’e ,ibsi…………. | |  |
| Kutaa 4ffa Gaaffilee waa’ee saala fayyaa waliin walqabatan | | | | |
|  | Yeroo ulfa turte walqunnamti saala taasifteta? | 1.eeyyee 2.lakkii |  | |
|  | Yoo eeyyee G401 jette hanga torbe meeqatti? | ---------------------Torbeedhan. |  | |
|  | Yoom eegalte walqunnamti saala da’umsa booda? | ---------------------Lakkoofsan |  | |
|  | Sababni kee maal fakkata kan ati walqunnamti kan eegalte? | 1.Gaaffiin abba warra kootin waan na dhiyaatef  2.Walii galteedhan  3.Kan biro yoota’e ,ibsi…………. |  | |
|  | Yeroo walqunnatti saala eegalte rakkoon qaama saala kee irra gahe jira | 1.eeyyee 2.lakkii |  | |
|  | Gaaffii 405 irratti deebiin kee yoo eeyye ta’efilanoo keessa isa kami?? | 1.Dhukkubi qaama saala  2.Tarsa’uu qaama saala  3.Dhangala’uu dhiiga qaama saalattin  4.Dhangalu xuriiqaamasaalatin  5.Fedha walqunnamtidhabu  6. Kan biro yoota’e ,ibsi…………. |  | |
|  | Gargarsa ogessa fayyaa argateetaa? | 1.Eeyyee 2.Lakki |  | |
|  | Barumsi ogessaa fayyaadhan si kennamera waa’ee jalqabi salqunnamti da’umsa booda? | 1.Naf kennamera  2.Naf hin kennamne |  | |

**BAAY’EE GALATOOMAA!**
