# Supplementary material for: Early resumption of postpartum sexual intercourse and its associated risk factors among married postpartum women who visited public hospitals of Jimma zone, Southwest Ethiopia: A cross-sectional study
Source: PLoS One. 2021 Mar 29;16(3):e0247769. doi: 10.1371/journal.pone.0247769 (PMC8007007; doi:10.1371/journal.pone.0247769)
Supplement: S1 File — (DOCX) [file pone.0247769.s002.docx]

**MEKELLE UNIVERSITY**

**COLLEGE OF HEALTH SCEINCES**

**DEPARTMENT OF MIDWIFERY**

**ANNEX ONE: PARTICIPANT INFORMATION SHEET**

**Introduction**

This questionnaire is prepared to assess the early resumption of sexual intercourse and factors associated among married postpartum women attending immunization centers at selected Jimma Public Hospitals. The assessment is made for the partial fulfillment of master’s degree in Clinical Midwifery. Your cooperation and participation until the completion of the questionnaire is very necessary for the successful completion of the assessment. We therefore ask your genuine willingness.

**Risk/ Discomfort**

By participating in this research project, you may feel that it has some discomfort especially on wasting time. But we hope you will participate in the study by considering the benefit of the research result. There is no risk or hazard in participating in this research project.

**Benefits**

If you participate in this research project, there may not be direct benefit to you. But the findings of this study will help us to identify the gap and take the appropriate intervention by the authorized stakeholder..

**Incentives**

You will not be provided any incentives or payment to take part in this project

**Right to refuse or withdraw:**

You have full right to refuse from participating in this research. You can choose not to respond to some or all questions if you do not want to give your response.

**Persons to contact:**

If you have any question to ask, please contact

Name : Tariku Bekela,cell phone:+251906180591 Email: gadaa2007@gmail.com
