# Supplementary material for: Early resumption of postpartum sexual intercourse and its associated risk factors among married postpartum women who visited public hospitals of Jimma zone, Southwest Ethiopia: A cross-sectional study
Source: PLoS One. 2021 Mar 29;16(3):e0247769. doi: 10.1371/journal.pone.0247769 (PMC8007007; doi:10.1371/journal.pone.0247769)
Supplement: Attachment — Submitted filename: Response to reviewer two.docx [file pone.0247769.s004.docx]

***Article Title: Early resumption of sexual intercourse and associated factors among married postpartum women at Jimma zone Public Hospitals, South West Ethiopia: A cross-sectional study***

1. **For the second reviewer & *many thanks for your suggestions, comments, and concerns***

**Here are a response to your comments and concerns**

1. The knowledge and advice gap on postpartum sexual health has been seen and modified.

So, the women must get adequate knowledge and advice on postpartum sexual health during the antenatal and postnatal period.

1. Paragraph 2, 3, and 4 one paragraph=the introduction section are rearranged and this paragraph also merged into one paragraph.

**Methods**

1. And most of the published articles were reported only on the timing of postpartum sexual intercourse resumption without considering its associated factors even though, knowing associated factors of postpartum sexual intercourse resumption are an important element to take an intervention". Cited by Reference No [5] and revised in the new paragraph.
2. The type of this study is facility-based cross-sectional and the consistency issue is corrected.
3. Code of ethics: the study participants were informed about the purpose, risks, and benefits of this study. And voluntary participation and no relation with the service given whether she participated in the study or not. Data collections were conducted after getting written informed consent from each participant. And for the participants aged below 18 years (to fit preset WHO criteria of the age) informed written consent was obtained from the parents or legal guardians.
4. Operational definitions and definitions of terms are omitted and written under the eligibility criteria and analysis section.

**Results**

1. Table**:1 & 2** merged into Table :**1** ,Table:**3 & 4**=merged in to Table=**2** and Table :**5** to Table**:3**
2. “It is interesting to note that, the slight decrease from the study done in Addis Ababa in the same locality compare to this study." Cited by Reference No [5]
3. The limitation of the study. “Lacking qualitative part of study design was other the limitation of this study because mother’s potential experience on intercourse resumption by culture, norms, and myths related factors were not exhaustively included”. Omitted and revised into being only a facility-based study was also among the limitation of this study. Finally, a causal relationship could not be assessed due to the cross-sectional design.

Thank you very much.

     With best regards!
