# Supplementary material for: Early resumption of postpartum sexual intercourse and its associated risk factors among married postpartum women who visited public hospitals of Jimma zone, Southwest Ethiopia: A cross-sectional study
Source: PLoS One. 2021 Mar 29;16(3):e0247769. doi: 10.1371/journal.pone.0247769 (PMC8007007; doi:10.1371/journal.pone.0247769)
Supplement: Attachment — Submitted filename: Response to Reviewers =new.docx [file pone.0247769.s005.docx]

***Article Title: Early resumption of sexual intercourse and associated factors among married postpartum women at Jimma zone Public Hospitals, South West Ethiopia: A cross-sectional study***

1. **Response to Reviewer One**

First, we want to appreciate your smart comments and suggestions, and said these here are our concerns and with some clarification on comments given:

**Generally comments**

1. Generally, comments on grammar, spelling, and subject-verb agreement= revised

      2. Yes, this study was done on mothers visited for vaccination of their child at 14 weeks.

- In the beginning, I intended to see a number of mothers resumed sexual intercourse during recommended and unrecommended period. We used 14 weeks because it is 3rd EPI schedule in Ethiopia and to access postpartum women easily.
- This study included mothers who visited immunization centers and women visiting other MCH services who were not approached by this study and we added them as a limitation of this study.

Methods sections.

- Many improvements have been done and the population also merged into one.

Result sections

- Amended

*Thank you very much.*

*With best regards!*

**2. For the second reviewer & *many thanks for your suggestions, comments, and concerns:***

- We have modified the language clarity issue accordingly.
- From the previous comments, we have made many changes to the document to have the PLOS-ONE journal format.

*Thank you in advance.*

*With best regards!*

**3.For the second reviewer.M*any thanks for your suggestions, comments, and concerns*.**

**As per the comments given, here are a response to your comments and concerns**

1. Comment 1: my evidence for the best time for sexual resumption is a published research article cited by [1,7,8,9,16,17 ].The best time to resume sexual resumption is after six weeks postpartum. It is a recommendation of many scholars, and also we included operational definitions in the methods part.
2. Yes, it is a sensitive topic but silent adverse health impact on the mother and child health also on the family at all. As you said, had it been a self-administered question, it was nice but this study is facility-based and almost 26% of participants are illiterate.
3. The reason why I took the 14 weeks rather than 6 weeks (2st EPI schedule in Ethiopia) is my intention at first was to see a percentage of women resumed during recommended vs unrecommended period. Balancing those resumed before and after six weeks. Also is selected because 14 weeks is the 3rd EPI schedule in Ethiopia to access all the postpartum women.

4. Sample size issue we used 73% proportion which was taken from the study done in Addis Ababa. This study is the most recent study done in Ethiopia.

5. P≤2 this cut of a point is taken from reference number [5]

6. Modified according to the comment give, and the place of residence is not mentioned under the table so we wrote it in the form text.

7. Modified accordingly

8. We revised the whole manuscript with language expertise.

9. The explanations issue was modified

10. Decimal point corrected throughout the document

11. Confidence interval issue, we will read it but still, we did not get the exact cut-off point for it.

12. This study is not free from recall bias. However, to minimize recall bias careful design was considered and data collectors asked them freely to remember it.

*Thank you very much.*

*With best regards!*
